# Helical tomotherapy in the radiotherapy treatment of Hodgkin's disease ‐ a feasibility study

**DOI:** 10.1120/jacmp.v11i1.3042

**Published:** 2010-01-28

**Authors:** Maria T. Vlachaki, Sanath Kumar

**Affiliations:** ^1^ Department of Radiation Oncology BC Cancer Agency‐Vancouver Island Centre Victoria BC Canada; ^2^ Department of Radiation Oncology Henry Ford Hospital Detroit MI USA

**Keywords:** 3D CRT, Helical TomoTherapy, Hodgkin's Disease

## Abstract

Radiation therapy for advanced Hodgkin's disease often requires large fields and may result in significant exposure of normal tissues to ionizing radiation. In long‐term survivors, this may increase the risk for late toxicity including secondary malignancies. 3D CRT has been successfully used to treat this disease but treatment delivery is often complex requiring matching of photon with electron beams, and utilization of field‐in‐field techniques and of partial transmission blocks. HT is an arc‐rotational intensity modulated radiation therapy technique proven to achieve superior target dose conformality and sharp dose gradients around critical normal tissues. HT, however, has also been associated with higher volumes of low‐dose regions in normal tissues and, therefore, higher integral dose. The present study was undertaken to compare the dosimetry of 3D CRT to HT in a pediatric patient with advanced HD. Clinical target volume (CTV) included bilateral lower cervical and supraclavicular areas, mediastinum, bilateral hili, left axilla and bilateral diaphragmatic lymph nodes. The planning target volume (PTV) was derived by circumferentially expanding the CTV by 1 cm. Whole lung and heart irradiation was also planned due to bilateral pleural and pericardial effusions. The prescribed radiation dose was 21 Gy to the PTV and 10.5 Gy to the whole lung and heart. Target coverage was comparable for both plans. The minimum, maximum, and mean PTV doses were 18.61 Gy, 22.45 Gy and 21.52 Gy with 3D CRT and 19.85 Gy, 22.36 Gy and 21.39 Gy with HT, respectively. HT decreased mean normal tissue dose by 21.6% and 20.07% for right and left breast, 20.40% for lung, 30.78% for heart, and 22.74% for the thyroid gland. Integral dose also decreased with HT by 46.50%. HT results in significant dosimetric gain related to normal tissue sparing compared to 3D CRT. Further studies are warranted to evaluate clinical applications of HT in patients with HD.

PACS number: 87.53.Kn

## I. INTRODUCTION

The treatment of Hodgkin's disease (HD) has evolved from using high‐dose extended field irradiation alone to combined modality therapy. Combination of multi‐agent chemotherapy with involved field radiotherapy is being increasingly used in the treatment of pediatric HD with excellent cure rates of 95% in early‐stage disease and 90% in advanced‐stage disease.^(^ ^1^ ^–^ ^4^ ^)^ With improved survival, long term effects and quality of life of survivors are becoming increasingly important issues.

There are various long‐term effects associated with the treatment of HD, especially in pediatric patients. These include second malignancies, cardiac toxicity, endocrine dysfunction, pulmonary disease, and growth impairment and may become apparent after ten or more years of observation.^(5)^ Risk factors include younger age, radiation therapy, chemotherapy, and time interval from treatment.^(^ ^6^ ^,^ ^7^ ^)^ With regard to radiation therapy, studies have demonstrated increased incidence of late effects with higher radiation dose and larger field sizes, especially subtotal and total nodal irradiation.^(^ ^6^ ^,^ ^8^ ^,^ ^9^ ^)^ Therefore, the utilization of techniques which minimize normal tissue radiation exposure may prove to be an effective treatment strategy in order to reduce the incidence of late toxicity in long term survivors with HD.

HT has been proven effective in achieving high‐dose gradients at the interface of tumor with surrounding normal tissues. Using intensity modulated radiation therapy (IMRT) treatment planning, it generates a spiral fan beam around the patient that is further modulated by a binary multileaf collimator.^(10)^ This results in a highly conformal dose distribution, especially in cases of targets located in proximity to critical normal tissues. As a result, HT has been used for stereotactic treatment of small tumors as well as for the treatment of larger targets such as in cranio‐spinal or total body irradiation.^(^ ^11^ ^–^ ^14^ ^)^

In this study, we present a dosimetric analysis comparing 3D CRT with HT plans in a pediatric patient with HD. Our hypothesis is that HT planning will result in reduced normal tissue radiation dose without compromise in target coverage.

## II. MATERIALS AND METHODS

### A. Case Presentation

The patient was a 16‐year‐old female with a history of Hodgkin's disease, nodular sclerosing subtype, stage IVB. The patient presented with supraclavicular, axillary and bulky mediastinal lymphadenopathy, bilateral pleural effusions, pericardial effusion and bilateral diaphragmatic lymphadenopathy. She was treated with chemotherapy followed by involved field radiation therapy (IFRT). Radiation therapy was administered according to the guidelines of Children's Oncology Group protocol AHOD0031.^(15)^ Chemotherapy consisted of Doxorubicin, Bleomycin, Vincristine and Etoposide. At completion of chemotherapy, repeat staging with contrast CT scan revealed a good partial response with significant decrease in the size of the mediastinal mass from 12.7 cm to 7.2 cm and complete resolution of adenopathy of the neck and axilla. There was a significant interval decrease in size of diaphragmatic lymph nodes and an interval resolution of pericardial and pleural effusions. The PET/CT (Positron Emission Tomography) scan demonstrated high Fluorodeoxyglucose (FDG) uptake at diagnosis which correlated with the sites of nodal involvement. Specifically, the highest FDG uptake was observed in the mediastinum at a maximum standardized uptake value (SUV) of 6.6. After completion of chemotherapy, only mild focal FDG activity remained in the mediastinum (SUV of 3.1). The patient received involved field radiation therapy four weeks after completion of chemotherapy.

### B. Treatment planning

Treatment planning was performed with CT scans of 5 mm thickness. In our study, 3D CRT treatment plan was generated using Eclipse treatment planning system (version 8.1) (Dentsply Ceramco, Inc., York, PA) using anisotropic analytic algorithm (AAA) (Varian Medical systems, Palo Alto, CA). The helical tomotherapy plan (TomoTherapy Inc, Madison, WI) was created using an inverse treatment planning system based on convolution/superposition algorithm (version 3.1).

### C. Target definition

Contouring was performed on the CT datasets using Eclipse treatment planning system and structures were subsequently transferred to the HT planning workstation using the DICOM RT protocol. For the definition of the target and organs at risk, the guidelines provided by the Children's Oncology group study AHOD0031 were utilized.^(15)^ The CTV incorporated the involved nodal regions as seen on CT and PET/CT scans obtained before chemotherapy except for mediastinal lymphadenopathy for which the postchemotherapy volume was used to define the radial boundaries of the tumor. PTV consisted of the CTV with 1 cm circumferential margin (nodal PTV). Bilateral lungs and heart were fused in a separate clinical target volume and were also given a circumferential 1 cm expansion (lung and heart PTV). For the 3D CRT treatment plan, an additional margin of 0.5–1 cm was given beyond the PTV to block edge to account for the penumbra. The “normal tissue” volume was defined as the whole patient volume minus the PTV. Organs at risk (OARs) considered in the present study include bilateral breasts, bilateral lungs, heart, thyroid gland, larynx, bilateral kidneys, liver, bilateral head of humeri, vertebral bodies, and spinal cord. The prescription dose to the nodal PTV was 21 Gy, while the prescription for the lung and heart PTV was 10.5 Gy.

### D. Three‐dimensional conformal radiation therapy (3D CRT)

**Figure 1:**
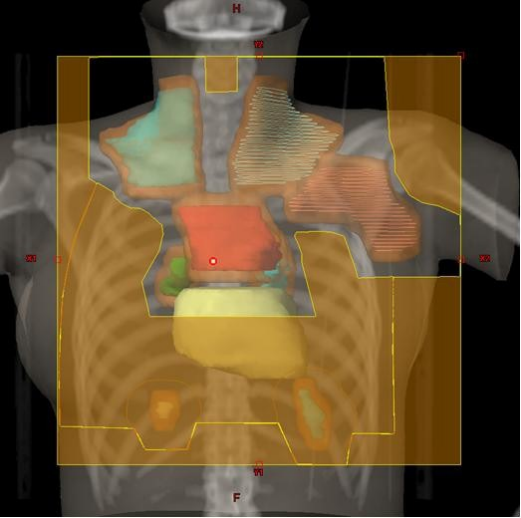
3D CRT plan setup with the cardiac silhouette demonstrating the involved nodal regions including bilateral supraclavicular, left axillary, mediastinal, bilateral hilar and diaphragmatic nodes. The PTV is depicted in brown color. A 50% transmission block was used to reduce the radiation dose to the lung and heart.

The patient was treated with 3D CRT technique using parallel opposed anterior‐posterior (AP) and posterior‐anterior (PA) fields and 6 MV photons. Using field‐in‐field technique, the dose at midplane throughout the treatment volume was maintained within the range of −5% to +7% of the prescribed dose, with point of maximum dose within the treated volume no more than 110% of the prescription dose. A 50% partial transmission block was used to reduce the total dose to bilateral lung and heart to 10.5 Gy. The disease in right and left diaphragmatic nodes was supplemented using appositional 12 MeV electron beams to a total dose of 21 Gy. Figure 1 demonstrates the PTV and radiation fields used for 3D CRT plan.

### E. Helical tomotherapy (HT)

The HT plan parameters were as follows: jaw setting, 2.5 cm; calculation grid size, 256×256; pitch, 0.30; modulation factor, 2. For tumor, the importance value was set at 100, and maximum and minimum dose penalties were set at 35 and 8, respectively. For organs at risk, the importance value was set at 1. The final plan values for importance, maximum and minimum dose penalties were adjusted to ensure that at least 95% of the PTV is covered by the prescribed dose of 21 Gy.

### F. Biological response models

Biological response models of tumor control probability (TCP) and normal tissue complication probability (NTCP) were calculated and compared for HT and 3D CRT plans.

For the TCP calculation, the Poisson model of tumor control was used.^(16)^ This is based on the assumption that tumor control is achieved when zero clonogenic cells survive the irradiation, and that the probability of this occurring obeys Poisson statistics. The probability (P) of 0 cells surviving given a mean surviving number N is given by:

(1) $$\text{TCP}=e(-\text{NSF}(D))=e^{-N{\left(SF_{2}\right)}^{\frac{D}{D_{\text{ref}}}\cdot \frac{{\;}^{\alpha }{/}_{\beta }+{\;}^{D}{/}_{n}}{{\;}^{\alpha }{/}_{\beta }+D_{\text{ref}}}}}$$

where N equals the total number of clonogen tumor cells, derived by multiplying the number of clonogen cells per cubic centimeter of CTV by the CTV volume. For the treatment of subclinical disease, the number of clonogen cells per cubic centimeter is assumed to be $10^{7}$.^(17)^ α/β=10Gy; where α and β are radiosensitivity parameters related to cell killing from single hit or multiple hit events, respectively. ${\text{SF}}_{2}$ is the surviving fraction after irradiation at a reference dose $D_{\text{ref}}$ of 2 Gy. In the present work, it is assumed that 50% of clonogenic cells survive after each irradiation fraction and, therefore, ${\text{SF}}_{2}=0.5$. D is the equivalent uniform dose EUD (Gy) defined as the radiation dose by which two different target dose distributions result in the same number of surviving clonogens and, therefore, in the same biological effect. In this equation, n equals number of treatment fractions.

NTCP values were calculated using the Lyman model^(18)^ and the Kutcher‐Burman reduction algorithm.^(19)^ The parameters used in the model ${\text{TD}}_{50},\;m$ and n have been tabulated by Burman et al. for different organs and specified end‐points.^(20)^

### G. Plan evaluation

The plans were compared with regard to target coverage as measured by the maximal, minimal, and mean PTV dose. The planning target volume receiving at least 95% of the prescription dose ${(V}_{95})$ and the dose covering at least 95% of the PTV ${(D}_{95})$ were also used for plan comparison. In addition, the target inhomogeneity index was calculated for each plan, and defined as the difference between the maximum and minimum PTV dose divided by the mean dose to the PTV.

The normal tissue dose was evaluated using the following criteria: the mean and maximum dose to OARs, the volume of OAR receiving 21 Gy ${(V}_{21})$, 15 Gy ${(V}_{15})$ and 10.5 Gy ${(V}_{10.5})$. The normal tissue (non‐tumor) integral dose (NTID) was also calculated for HT and 3D CRT plans as a product of mean dose multiplied by the volume of the structure outside the target.^(21)^

## III. RESULTS

**Figure 2:**
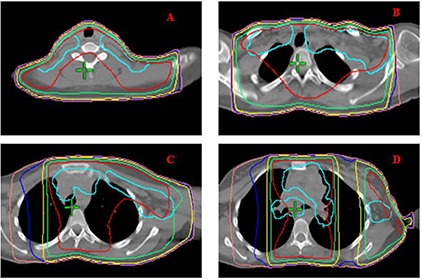
3D CRT isodose distribution of representative axial slices. PTV is shown in cyan. Red, green, yellow, blue, and pink isodose lines represent 21, 19.6, 15, 12, and 10 Gy, respectively.

**Figure 3:**
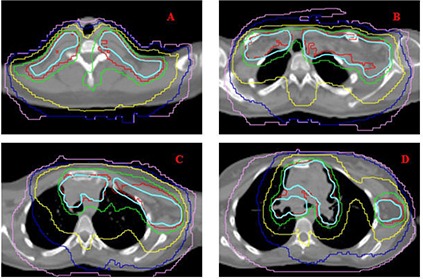
HT isodose distribution of the same axial slices presented in Fig. 2. PTV is shown in cyan. Red, green, yellow, blue, and pink isodose lines represent 21, 19.6, 15, 12, and 10 Gy, respectively.

**Figure 4:**
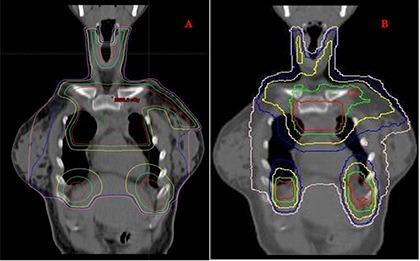
Coronal view showing isodose distributions for a 3D CRT plan (A) and HT plan (B). Red, green, yellow, blue, and pink isodose lines represent 21, 19.6, 15, 12, and 10 Gy, respectively.

Figure 1 demonstrates radiation fields with 3D CRT. Figures 2 and 3 demonstrate the isodose distributions for 3D CRT and HT, respectively, at four different axial levels. Figure 4 demonstrates the isodose distribution in coronal plane for 3D CRT and HT. The target coverage was comparable for both 3D CRT and HT plans. The minimum, maximum, and mean PTV doses were 18.61 Gy, 22.45 Gy and 21.52 Gy with 3D CRT, and 19.85 Gy, 22.36 Gy and 21.39 Gy with HT, respectively. The percentage of PTV receiving at least 95% of the prescription dose ${(V}_{95})$ was 100% and 99.7% for 3D CRT and HT plan, respectively. Also, the dose covering at least 95% of the PTV ${(D}_{95})$ was 20.78 Gy for the 3D CRT plan compared to 20.98 Gy for the HT plan. The dose inhomogeneity index was 0.18 for the 3D CRT plan compared to 0.12 for the HT plan. The calculated TCP values were 93.68% and 94.13% with 3D CRT and HT plans, respectively. The minimum, maximum, and mean lung and heart PTV doses were 10.27 Gy, 22.55 Gy and 16.42 Gy with 3D CRT, and 9.88 Gy, 22.13 Gy and 13.23 Gy with HT, respectively. The lung and heart PTV $V_{95}$ was 100% for 3D CRT and 98.94% for HT, while the $D_{95}$ was 11.79 Gy and 10.54 Gy for 3D CRT and HT, respectively. Figure 5(a) demonstrates the DVHs for nodal PTV and lung and heart PTV with 3D CRT and HT plans.

**Figure 5(a):**
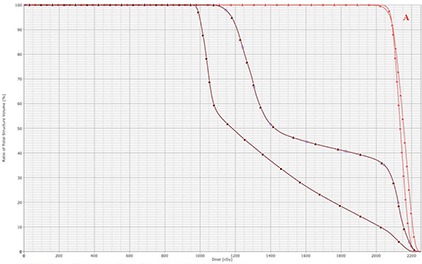
DVHs of nodal PTV (red) and lung and heart PTV (brown) with 3D CRT (triangle) and HT (square).

Compared to the 3D CRT plan, the HT plan resulted in a decrease of minimum, maximum, and mean dose to OARs (Table 1. Specifically, mean dose to right and left breast decreased from 9.85 Gy and 14 Gy with 3D CRT to 7.72 Gy and 11.19 Gy with HT, respectively. Similarly, mean dose to the thyroid gland, the lungs and heart were 22.74%, 20.40%, and 30.78% lower with the HT plan. HT delivered slightly higher dose only to the shoulders. The mean doses to the right and left shoulders were 1.47 Gy and 8.74 Gy with HT compared to 1.38 Gy and 2.71 Gy with 3D CRT planning, respectively. In addition, $V_{21},V_{15}$ and $V_{10.5}$ decreased with the HT plan compared to the 3D CRT plan (Table 2. Specifically, $V_{21}$ for right and left breasts were 2.24% and 13% with 3D CRT and 0% and 1.5% with HT, respectively. Similarly, $V_{21}$ for lungs with was 22.30% with 3D CRT and 3.78% with HT, respectively. Figure 5(b) demonstrates the DVHs for different OARs with 3D CRT and HT plans.

**Table 1:** Minimum, maximum, and mean doses for different organs at risk using 3D CRT and HT plans.

	*Minimum Dose (Gy)*		*Maximum Dose (Gy)*		*Mean Dose (Gy)*	
	*3D CRT*	*HT*	*3D CRT*	*HT*	*3D CRT*	*HT*
Right Breast	0.80	3.32	21.95	20.47	9.85	7.72
Left Breast	1.46	4.42	22.22	21.67	14	11.19
Liver	0	0.38	21.98	21.76	10.5	7.2
Heart	12.63	7.48	22.27	21.17	16.44	11.38
Lung	11.06	7.52	22.35	21.99	16.22	12.91
Thyroid	18.23	8.52	21.94	21.67	21.15	16.34
Larynx	7.58	5.77	20.94	12.19	12	8.25
Vertebral Body	1.48	0.35	22.26	20.69	14.72	9.61
Spinal Cord	1.52	0.35	22.26	20.69	14.72	9.61
Right Kidney	1.35	0.40	7.94	5.48	3.33	2.18
Left Kidney	1.52	0.39	13.43	5.7	4.42	2.3
Right Shoulder	1.17	0.92	1.85	4.20	1.38	1.47
Left Shoulder	1.25	4.39	17	18.59	2.71	8.74

**Table 2:** Percent volume of organs at risk receiving $10.5\;\text{Gy}\;(V_{10.5}),15\;\text{Gy}\;(V_{15})$, and 21 Gy ${(V}_{21})$ using 3D CRT and HT plans.

*OARs*	$V_{10.5}(\%)$		$V_{15}(\%)$		$V_{21}(\%)$	
	*3D CRT*	*HT*	*3D CRT*	*HT*	*3D CRT*	*HT*
Right Breast	59.98	17.26	9.53	2.81	2.24	0
Left Breast	81.08	51.30	38	22.7	13	1.5
Liver	64.92	34.38	10.90	3.09	0.90	1.55
Heart	100	81.3	47.06	24.7	27.20	0
Lung	100	85	46.11	34.3	22.30	3.78
Thyroid	100	93.3	100	61.59	69.72	8.81
Larynx	45.57	5.28	28.45	0	1.18	0
Vertebral Body	77	54.56	45.40	27.72	41.60	0.58
Spinal Cord	72.30	49.69	48.84	32.94	38.88	0
Right Shoulder	0	0	0	0	0	0
Left Shoulder	0.51	25.80	0.03	4.67	0	0

**Figure 5(b):**
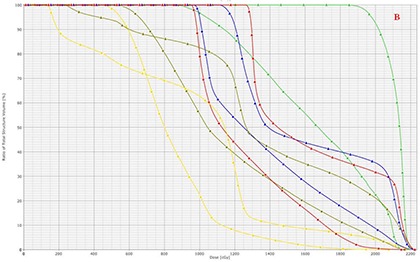
DVHs of OARs with 3D CRT (triangle) and HT (square). The OARs shown include right breast (yellow), left breast (dark green), lung (blue), heart (red), and thyroid (light green).

NTCP values were zero for all OARs in both plans, except for lung for which the NTCP was 0.1 with 3D CRT and 0.07 with HT, respectively. NTID was also decreased with HT by 46.50%, from 181.7 liter‐Gy to 96.58 liter‐Gy.

## IV. DISCUSSION

Although high cure rates have been reported in patients with HD, concerns have been raised regarding long‐term toxicity and quality of life among survivors.^(7)^ With prolonged life expectancy and follow‐up, the risks and potential impact of late toxicity are becoming increasingly important factors in determining treatment decisions. Second malignancies, cardiovascular disease, and pulmonary toxicity are among those late effects observed in long‐term survivors of HD.^(^ ^5^ ^,^ ^22^ ^)^

There is significant correlation between radiation dose and field size with the development of secondary cancer.^(^ ^8^ ^,^ ^9^ ^,^ ^23^ ^)^ With increased use of chemotherapy, high radiation doses have been replaced by low‐dose regimens. In addition, radiation volumes have also gradually decreased from subtotal or total nodal irradiation to IFRT. Nevertheless, a significant amount of normal tissue is still exposed to radiation even with IFRT, with the majority of second malignancies arising within or at the edges of irradiated areas.^(9)^

In our case, 3D CRT and HT produced comparable target coverage, while HT resulted in significant reduction in normal tissue radiation dose and integral dose. For the breast tissue specifically, HT decreased $V_{10.5}$ from 59.98% to 17.26% and from 81.08% to 51.3% for the right and left breast, respectively. Published studies have shown that the relative risk for breast cancer is dose‐dependent, increasing from two‐ to eight‐fold when the radiation dose delivered to breast tissue increases from 4 Gy to 40 Gy.^(24)^ In addition, dose reduction from 35 Gy to 20 Gy results in 40% breast cancer risk reduction in patients treated with IFRT.^(25)^ The same relationship between radiation dose and the development of secondary malignancies has been observed for other secondary cancers including lung and thyroid.^(^ ^26^ ^,^ ^27^ ^)^

Radiation‐induced heart disease accounts for increased deaths in long‐term survivors of HD.^(28)^ Published data indicate that doses as low as 15 Gy result in a 2.5% risk of cardiac toxicity at five years.^(29)^ Therefore, radiotherapy techniques that minimize cardiac exposure may prove effective in minimizing cardiac injury. In comparison to 3D CRT in this study, HT reduced mean heart dose by an additional 30.78%, and $V_{21}$ from 27.20% to 0%.

Radiation pneumonitis is another serious complication following thoracic irradiation for HD. In a study of adult patients with HD, the risk of pneumonitis was 11% or higher, and was observed with mean lung dose of 14 Gy and $V_{20}$ of 36% or more.^(30)^ In our patient, HT significantly decreased $V_{15}$ and $V_{21}$ from 46.11% and 22.30% to 34.3% and 3.78% with 3D CRT, respectively. HT also decreased lung NTCP by 30% from 0.1 to 0.07. This favorable dosimetric profile of HT may therefore limit pulmonary complications in HD patients treated with supradiagphragmatic irradiation.

In our study, the shoulders were the only tissues receiving minimally higher radiation dose with HT. Review of the literature indicates a steep radiation dose‐effect relationship for epiphyseal growth impairment between radiation dose of 15 Gy and 30 Gy, and doses in excess of 25–33 Gy have been associated with significant bone growth impairment.^(31)^ In our case, bone growth impairment is an unlikely long‐term sequela with either of the plans since the dose to both shoulders falls below the threshold for radiation‐induced bone damage.

Although IMRT is known to achieve higher target dose conformality and superior sparing of normal tissues, its use in HD has been limited to cases of massive mediastinal disease or those requiring re‐irradiation.^(32)^ Compared to 3D CRT, IMRT and HT have been associated with higher integral dose due to larger normal tissue volume exposure to low‐dose radiation. Consequently, higher risk for secondary malignancies has been estimated for IMRT.^(33)^ In our patient, however, 3D CRT resulted in higher integral dose by 46.50%, as significant normal tissue volume was within the portals irradiating the entire lung and heart.^(14)^

Complex treatment plans with 3D CRT require radiation field matching which inherently carries a risk for errors in the implementation of treatment. In our case, we matched photon fields with 100% and 50% partial transmission blocks with electron fields for the treatment of diaphragmatic lymph nodes. Electron beams are known to generate dose uncertainties at the edge of the radiation field with the high‐dose isodose lines bowing inside and the lower dose isodose lines bowing outside of the geometrical edges of the field. These uncertainties become more pronounced when electron fields are placed adjacent to photon fields and have the potential of underdosing the target or, conversely, overdosing the surrounding normal tissues. With their ability to modulate the radiation beams, IMRT and HT obviate the need for electron beams or field matching without compromising on target and normal tissue dose constraints.

In designing radiation treatments which include lung and heart, consideration has to be given to the potential motion of these tissues during radiation delivery. In this case, we did use a CTV expansion of 1 cm around the involved lymph nodes, lung, and heart, ensuring coverage of the diaphragmatic recesses bilaterally. Recent work by Girinsky et al.^(34)^ presented the concept of treating the prechemotherapy nodal disease with 1 cm margin. The rationale for this treatment approach is supported by the patterns of failure in HD which predominantly occur in the originally affected lymph nodes.^(35)^ Such narrow margins also minimize radiation exposure of normal tissues and have already been adopted for the design of new randomized trials by the European Organization for Research and Treatment of Cancer (EORTC) and Groupe d'Étude des Lymphomes de l'Adulte (GELA).

With highly conformal techniques, the use of image‐guided radiation therapy is highly advised for patient and tumor position verification at the time of treatment. HT offers image‐guided radiation therapy capability for patient and target position verification through the acquisition of megavoltage computerized tomography images right before each radiation treatment. However, movements of lung and other thoracic contents may sometimes exceed 1 cm, especially when located close to the diaphragm.^(36)^ Therefore, additional planning using four‐dimensional gated CT may prove useful in evaluating the degree of tumor and lung motion in patients with HD, and allow for adjustment of treatment margins in three dimensions.^(^ ^37^ ^,^ ^38^ ^)^ Moreover, techniques such as abdominal compression may also prove useful as they minimize diaphragmatic motion during the various phases of respiration.^(39)^ Finally, treatment planning of large fields with HT may lead to significant tomotherapy thread effect.^(40)^ Although a pitch size of 0.30 was used in this case, dose homogeneity may improve more by utilizing smaller pitch or double threading. Such strategies will further individualize treatment planning in order to optimize normal tissue protection without compromising on target coverage.

## V. CONCLUSIONS

In conclusion, HT resulted in comparable target coverage and better normal tissue sparing compared to 3D CRT in our HD patient. Further clinical studies are under way to validate this technique and evaluate clinical outcomes regarding tumor control and late toxicities. Routine use of gated techniques to monitor respiratory motion and image‐guided radiation therapy to verify tumor/patient positioning will be indispensable in ensuring accuracy in target coverage and maximum avoidance of surrounding healthy tissues.
